# A review of recent evidence relating to sugars, insulin resistance and diabetes

**DOI:** 10.1007/s00394-016-1340-8

**Published:** 2016-11-23

**Authors:** I. A. Macdonald

**Affiliations:** Queen’s Medical Centre, School of Life Sciences, University of Nottingham Medical School, University of Nottingham, Clifton Boulevard, Nottingham, NG7 2UH UK

**Keywords:** Sugars, Sucrose, Fructose, Glucose, HFCS, Insulin resistance, Diabetes mellitus

## Abstract

The potential impact on health of diets rich in free sugars, and particularly fructose, is of major concern. The focus of this review is the impact of these sugars on insulin resistance and obesity, and the associated risk of developing type 2 diabetes. Much of the concern is focussed on specific metabolic effects of fructose, which are argued to lead to increased fat deposition in the liver and skeletal muscle with subsequent insulin resistance and increased risk of diabetes. However, much of the evidence underpinning these arguments is based on animal studies involving very large intakes of the free sugars. Recent human studies, in the past 5 years, provide a rather different picture, with a clear dose response link between fructose intake and metabolic changes. In particular, the most marked effects are observed when a high sugars intake is accompanied by an excess energy intake. This does not mean that a high intake of free sugars does not have any detrimental impact on health, but rather that such an effect seems more likely to be a result of the high sugars intake increasing the chances of an excessive energy intake rather than it leading to a direct detrimental effect on metabolism.

## Introduction

There is increasing interest in the possibility that a high intake of refined sugars is associated with detrimental effects on metabolism that increase the risk of cardiovascular disease, obesity, insulin resistance and diabetes. Some of the evidence underpinning these concerns is derived from cross-sectional surveys or ecological observations, but there are also prospective cohort studies and controlled intervention trials which have addressed this issue. This brief narrative review arises from a Symposium on Sugars and Health held at the European Nutrition Conference in October 2015. It will consider the recent work, published in the past 5 years, which has focussed on the potential impact of dietary sugars on health, in particular insulin resistance and diabetes risk, and seeks to identify the intakes which might be associated with health-related problems. The terminology employed in these discussions is often used rather loosely, with the term ‘sugar’ often being used to represent a range of different molecules. For the purposes of this article, ‘sugar’ is taken to be the common term for sucrose, and the collective term ‘sugars’ will be used to include sucrose, glucose and fructose together with the high fructose corn syrups which are replacing sucrose in sweetened beverages and foods in many countries.

## Basic aspects of dietary carbohydrates, insulin action and insulin resistance

Dietary carbohydrates include the monosaccharides, disaccharides, oligosaccharides (chain length 3–9 molecules), polysaccharides and fibre. The monosaccharides are glucose, fructose and galactose, and the major disaccharides are sucrose (glucose plus fructose), lactose (glucose and galactose) and maltose (2 glucose molecules). The polysaccharide category represents the starches, which are made up of glucose polymers with variation in the location and number of cross-linkages between the molecules producing different sub-categories of starches. The mono- and disaccharides are normally combined into the collective category of sugars. The main carbohydrate contributors to dietary energy intake are the sugars and starches. The control of carbohydrate metabolism is dependent on appropriate levels of insulin secretion and insulin action. For the purposes of the present overview, the issues around insulin secretion (particularly as it relates to type 2 DM) will not be considered. However, the impact of dietary carbohydrates or total energy intake on insulin action is worthy of consideration.

Whilst there is currently some controversy over the role of carbohydrates in a healthy diet, most if not all national guidelines recommend that the proportion of energy contributed by carbohydrate should be approximately 50% (e.g. [1]). It has been known for at least 80 years that a low carbohydrate/high fat intake is associated with poorer glucose tolerance and insulin resistance [2]. The major concern at present is focussed on dietary sugars, and whether an excessive intake of them as a proportion of total carbohydrate can lead to insulin resistance and impaired glucose tolerance and lead to an increased risk of developing type 2 diabetes mellitus (DM). There is increasing concern about the potential threat to health represented by a high intake of sugars, as evidenced by the recent Dietary Guidelines for Americans [3], UK Scientific Advisory Committee for Nutrition’s Carbohydrate report [1] and the WHO Sugars Report [4]. Although most of the attention has been focussed on sucrose and fructose, many studies have failed to directly compare fructose and glucose in randomised trials, and when direct comparisons have been made, it is clear that any impact on health is more likely to be a sugars-related effect than specifically due to fructose (see later).This short review will consider this issue in greater detail and assess the potential problems of sugars in relation to insulin resistance and risk of type 2 DM. The present article is a narrative review based on the systematic reviews undertaken to inform the SACN Review of Carbohydrates and Health. The details of these 3 systematic reviews are provided in the SACN report, including the search strategies and the inclusion/exclusion criteria. The additional literature included in the present review was identified separately from this systematic review process, and this was not performed using systematic review criteria but was simply designed to illustrate some of the additional aspects of this field and the more recent observations reported in the past 5 years. One of the major issues in this area of research is the short-term nature of many of the investigations, especially studies involving alteration of the carbohydrate composition of the diet or the types of sugars being consumed. Clearly the long-term health effects of such interventions can only be speculated about, but such studies do provide useful information on potential mechanisms of effects of carbohydrates on health.

## What are the major issues relating to dietary fructose? Contrast between research evidence and speculation/opinion

There has been concern expressed for many years that sucrose, and particularly fructose, represent potential problems as far as risks to health are concerned. One of the first major critics of sucrose in the diet was John Yudkin [5], whose book ‘Pure, white and deadly’ summarised the scientific information available in the 1960s/early 1970s and promoted his opinion conveyed in the book’s title that diabetes, cardiovascular disease and other chronic illnesses were contributed to by a high sucrose intake. In more recent times, these views have resurfaced and been extended to include the high fructose corn syrup (HFCS) content of foods and drinks. These opinions relating to the threat to health from fructose alone and as a component of sucrose and HFCS, are sustained despite reports such as that from the German Nutrition Society, which concluded that intakes of up to 100 g fructose per day are not associated with increased serum triglycerides and that fructose and sucrose are not associated with increased risk of hypertension or CHD [6].

The principal concern raised in relation to fructose is that its metabolism promotes lipid synthesis in the liver and because it is metabolised independently of insulin action in the liver and elsewhere, it can lead to excessive lipid formation and deposition, insulin resistance and other features of the metabolic syndrome. However, the studies which have investigated potential effects of fructose on liver fat content, plasma lipids or insulin sensitivity have often failed to include a glucose control treatment, and thus any effect ascribed to fructose may simply be an effect of monosaccharides (see below). Longitudinal cohort studies and ecological observations have been used to associate increased obesity with high levels of sucrose, fructose or HFCS consumption. For example, Fiorito et al. [7] reported that higher sweetened beverage intake in 5-year-old girls was associated with increased BMI and adiposity 10 years later, whilst Nissinen et al. [8] in the young Finns study reported an association between increasing sugar-sweetened beverage intake from childhood to adulthood was associated with increased adult BMI in women. However, such observations can at best signal a concern that then needs investigating in controlled trials.

In some cases, the arguments for an increased risk to health associated with high intake of fructose arise from reviews of experimental animal studies combined with human ecological observations. An example of this is provided by Johnson et al. [9] who reviewed studies in metabolic syndrome prone rats that developed fatty livers whilst receiving 40% of their dietary energy from sucrose. This review also attributed to Lustig and colleagues [10], showing an epidemiological association of increased diabetes prevalence with increased sugar availability. However, this was a population level econometric analysis based on multiple cross-sectional observations. It is rather speculative and undesirable to draw major conclusions of causation or association from such a combination of econometric and animal data.

There is evidence from other animal experiments of effects of sucrose or fructose plus glucose on insulin resistance, liver fat content and liver levels of inflammatory markers [11]. However, these rats received 60% of their total energy intake as sucrose, or 30% as fructose and 30% as glucose. Both of these dietary treatments are so extreme; it is not surprising that metabolic abnormalities were observed. Another study fed mice with a diet providing 38.5% of energy as sucrose and observed elevated plasma glucose levels in the early phase of an oral glucose tolerance test (OGTT) indicative of insulin resistance or impaired insulin release [12]. However, the same study showed that sucrose feeding improved insulin sensitivity as assessed during an insulin tolerance test. One possible explanation of these apparently opposing observations is that high levels of sucrose feeding to these mice may have led to a reduction in the release of one or more of the incretin hormones from the GI tract during food/glucose ingestion, reducing the insulin response to the OGTT and thus leading to higher plasma glucose levels.

## What is the evidence from randomised controlled trials (RCT) of the metabolic effects of fructose or sucrose?

Some reviews and commentaries have urged caution in attributing major health risks associated with fructose/sucrose in people. Klurfeld et al. [13] assessed the potential link between HFCS and obesity and concluded that the evidence was not supportive of such a claim and commented that RCT at levels even exceeding normal human consumption have also been inconclusive related to fructose, sucrose and obesity. A subsequent review by Tappy and Mittendorfer [14] commented that whilst some studies describe potential adverse effects of high fructose intakes, particularly as part of an excessive energy intake, there does not appear to be a significant detrimental effect of fructose when part of a weight-maintaining diet. They concluded by commenting that definitive studies were missing. Since then, a number of RCT have been published which shed further light on this issue.

Bravo et al. [15] performed a study in healthy, overweight adults which involved providing sweetened low-fat milk which contained 8, 18 or 30% of estimated daily energy requirements in the form of sucrose or HFCS, for a period of 10 weeks. There was an increase in reported energy intake over the course of the study when all groups were combined, and whilst the groups receiving 18 and 30% of energy from the sugars appeared to have the most marked increases, there were no significant increases in any group. There was an increase in body weight across the study, although this was only significant in the 30% sugars groups with mean increases of 1 and 2.3 kg over 10 weeks in the 30% sucrose and the 30% HFCS groups. Despite the increase in body weight, there were no significant changes in the fat content of the liver or skeletal muscles in any group. When data for all groups were combined, there was a significant increase in plasma triglycerides (from 1.11 to 1.27 mmol/l), but there were no differences between the groups and no significant change within any individual group (presumably due to a lack of statistical power). There were no changes in plasma cholesterol during the study.

Heden et al. [16] also found no effect of moderate daily intakes of mixtures of fructose and glucose for a period of 2 weeks in physically active adolescents. They consumed mixtures containing 50 g fructose and 15 g glucose, or 15 g fructose and 50 g glucose, daily for 2 separate 2-week periods. When these supplements were considered together with the dietary fructose and glucose, the total intakes were around 70 g fructose and 40 g glucose, compared to 40 g fructose and 70 g glucose. The combined sugars intakes equated to approximately 25% of total energy intake. There were no significant changes in fasting and postprandial indices of insulin resistance, or fasting blood lipids with either treatment, or any difference in response between subjects who were overweight compared to the healthy weight subjects.

An interesting study by Aeberli et al. [17] assessed the effect of different amounts of fructose or glucose supplementation in a beverage, on insulin sensitivity using the glucose clamp technique. Intriguingly, the title of the paper is ‘Moderate Amounts of Fructose Consumption Impair Insulin Sensitivity in Healthy Young Men’, but the results obtained show that the only change in insulin sensitivity was a reduction in the degree of insulin-induced suppression of hepatic glucose production after the high fructose drink (80 g/day for 3 weeks) intake compared with the 80 g glucose drink. There were no differences in whole body, systemic insulin sensitivity and no differences between the groups in fasting insulin and glucose concentrations. Furthermore, there was no difference in any component of insulin sensitivity between the moderate fructose (40 g/day) drink or 80 g sucrose drink and the 80 g glucose control drink. This suggests a threshold intake of fructose is needed for an effect on hepatic insulin resistance. The total fructose intake after the 40 g drink was 77 g/day, whilst the total fructose intake after the 80 g drink was 115 g/day. Thus, from this study the threshold appears to be somewhere between fructose intakes equivalent to 15 and 23% of total energy intake. It is interesting to note that there did not appear to be any impact of these sugars containing drinks on total energy intake compared to the baseline condition, with some compensatory reductions in protein and fat intake balancing the additional energy from sugars. Such compensation has not been reported in other studies involving increased intake of sugars containing drinks. There was also no effect on fasting serum triglycerides which is surprising as other studies have shown an increased monosaccharide intake leads to a rise in triglycerides.

An interesting contrast to the Aeberli study is found in the work of Hokayem et al. [18] who studied insulin resistance in relatives of people with type 2 DM. Because of their family history, such people have an increased risk of developing diabetes and are likely to be insulin resistant. The purpose of this study was to examine the effects of grape polyphenols on the metabolic consequences of short-term consumption of a high fructose intake (3 g fructose/kg fat free mass per day for 6 days). The fructose intake was designed to induce insulin resistance and oxidative stress. The group consuming the grape polyphenols did not show any reduction in glucose infusion rate during the glucose clamp or any change in the fasting insulin sensitivity index after 6 days of fructose consumption. By comparison, the placebo control group had an 11% reduction in glucose infusion rate and a 19% reduction in the fasting insulin sensitivity index.

Stanhope et al. [19] provided obese subjects with 25% of their energy requirements as glucose or fructose for 10 weeks. Both groups had similar increases in body weight (+1.8% in the glucose and +1.4% in the fructose groups) and body fat content (+3.2 and +2.8%), indicating that this was essentially an overfed state. Only the fructose group showed an increase in postprandial serum triglycerides (TG) from late afternoon onwards, with no change in fasting TG in either group. There was also evidence of impaired glucose tolerance with increased insulin responses in the fructose group, with no change in the glucose group. However, the oral glucose load in the glucose tolerance test is not something that the fructose group would have been used over the previous weeks, and so without glucose clamp-based assessments of insulin resistance, it is difficult to interpret the glucose tolerance data.

Lecoultre et al. [20] reported a study of the effects of fructose, glucose or fat overfeeding for 6 days on hepatic insulin sensitivity and intrahepatic lipids in healthy people. Three different doses of fructose were investigated, 1.5, 3 and 4 g per kg body weight per day, which were additional to the diet required to maintain weight. This equated to an excess energy intake of 17, 32 and 43% of energy requirements. The glucose dose used was 3 g/kg/day (37% overfeeding), and the fat overfeeding was at a level of 30% of energy requirements. The 3 and 4 g doses of fructose, the glucose and the fat overfeeding all increased liver fat content by 50 to 110%, with the 3 g fructose dose having a significantly larger effect that the 3 g glucose. Interestingly, the smaller degree of overfeeding with 1.5 g fructose did not increase liver fat content. The two higher doses of fructose (but not 1.5 g fructose or the glucose or fat overfeeding) also reduced the hepatic insulin sensitivity by about 20%. Thus, high fructose intakes during overfeeding did increase liver fat and produce liver insulin resistance, but 100 g of fructose per day (the lowest dose) with modest overfeeding did not have such effects.

Stanhope et al. [21] recently reported a study of the effects of varying amounts of high fructose corn syrup (HFCS) consumed for 2 weeks, with the lowest amount being equivalent to 10% of energy requirements. This lowest quantity represents approximately 5% of total energy from fructose and was not associated with any change in body weight over the 2 weeks, whilst the 17.5 and 25% HFCS groups did have an increase in weight. The 17.5 and 25% doses of HFCS were accompanied by increases in postprandial TG, and the 25% dose also tended to increase fasting TG. Interestingly, the 10% HFCS treatment also increased postprandial TG in the late evening, indicating that this effect is not dependent on an increase in body weight. No data were presented on glucose and insulin, so it is not possible to comment on any effects on insulin resistance of this low dose of HFCS.

The overall conclusion from these studies is that a fructose intake exceeding 150 g/day in adults reduces fasting insulin sensitivity but intakes appear to need to exceed 250 g/day before affecting insulin-induced suppression of liver glucose output. There is less clarity of potential effects on peripheral insulin sensitivity, even at high fructose intakes. The higher doses of fructose also appear to increase serum TG, in at least some studies, and it is possible that this can occur with low doses of fructose in so far as late evening postprandial TG are concerned and without an increase in body weight (i.e. without overfeeding). It is striking that the majority of these observations are linked to situations where high fructose intakes are accompanied by excessive energy intakes, and the following section considers this in more detail.

## Is the effect of fructose or sucrose affected by the amount consumed or the total energy intake?

A study of ours directly compared high levels of glucose or fructose intake in overweight, large-waisted men who were otherwise healthy [22]. The participants were studied in two separate 2-week periods, the first when fed at energy balance (isoenergetic), with glucose or fructose providing 25% of total energy intake, and the second, at least 6 weeks later, when the sugars were added to their ad libitum diet (with a target of 25% overfeeding). The men had high initial levels of liver (8%) fat and intramuscular (8%) fat at baseline, but there were no significant changes in either the fructose or glucose groups when an isoenergetic diet was provided. There was an increase in fasting insulin resistance in the fructose group (HOMA-IR increased from 3.6 to 4.3) but not the glucose group (3.2 to 3.4), but there were no significant changes in the suppression of hepatic glucose output or peripheral insulin sensitivity during a glucose clamp in either group. By contrast, when the same subjects were overfed, there were increases in liver fat content to approximately 10% in both the fructose and glucose groups, together with a trend for an increase in muscle fat of a similar amount. With this fructose or glucose overfeeding, there were no changes in HOMA-IR, and also no significant changes in suppression of hepatic glucose production or peripheral insulin sensitivity during the glucose clamp, although there was a trend for a reduction in the latter which was more noticeable in the glucose-treated group. The mean doses of fructose or glucose provided in this study were around 210 g/day, which is consistent with the previous section in which it was suggested that fructose intakes need to exceed 150 g/day before potentially deleterious effects are observed. The other conclusions from this study are that in overweight men with an elevated liver fat content, these effects are similar for fructose and glucose and are predominantly linked to overfeeding than the sugars per se [22].

This link between excessive energy intake and deleterious effects of sugars is also illustrated by the review by Te Morenga et al. [23], which formed part of the basis for the WHO Sugars and Health report [4]. This systematic review clearly showed that higher sugars intakes were linked to increased body weight or fatness if ad libitum diets were considered, but if sugars were exchanged for other carbohydrates and energy intake was maintained constant, then there were no deleterious effects of sugars.

A similar conclusion arose from the SACN Carbohydrates and Health Report [1], with higher sugars intake in an ad libitum situation being associated with higher energy intake. This report provided a meta-analysis of the link between change in sugars intake and change in energy intake in ad libitum intake in randomised controlled trials which showed that the higher sugars intake (with a mean increase of 10%) was associated with an increased energy intake of approximately 1 MJ/day. Whether this represents an increased risk of developing type 2 DM, or just an increased risk of weight gain and eventually obesity, cannot be determined from these short-term RCT.

It is interesting to note that the SACN report also looked at cohort studies examining associations between sugars intake and incidence of type 2 DM. Whilst the studies identified had too much heterogeneity to allow a meta-analysis to be performed, it was notable that they provided no consistent evidence of an association between diets differing in the proportion of sugars and the incidence of type 2 diabetes mellitus.

A balanced overview of the current position regarding potential health problems arising from fructose/sucrose was provided by van Buul et al. [24]. They concluded that fructose as normally consumed in foods does not exert specific metabolic effects that would contribute to weight gain. They also concluded that the specific problems which have been identified in relation to sugars-sweetened beverages and risk of obesity are a result of energy overconsumption rather than any effect of fructose on energy metabolism or storage. However, it is clear that limiting sugars intake as part of a strategy to limit energy intake whilst at the same time increasing physical activity to increase energy expenditure, represent the lifestyle changes needed to address the current obesity problem in many countries. Clearly, the recent observation of potential effects of modest amounts of HFCS on postprandial TG, without associated weight gain, needs to be recognised [21]. However, it is worth noting that the 10% HFCS that was consumed is double the recommended intake of 5% of energy from free sugars which was concluded in the SACN report.

## Should we be more concerned about dietary glycaemic characteristics than the sugars content?

There is increasing interest in the possibility that the glycaemic characteristics of carbohydrates may also be of importance in relation to optimal health. The original concept of glycaemic index was developed to help people with diabetes to improve their glucose control. In recent times, it has gained a wider application, and assessing the glycaemic index and glycaemic load of diets has been included in a number of prospective cohort studies which are summarised in the SACN report [1]. This has led to several randomised controlled trials of potential benefits of lower glycaemic index diets, including a recent study of our own (Bawden et al. [25]) which showed that healthy non-obese young men consuming a high glycaemic index diet for 7 days showed an increase in liver fat content, whereas 7 days on a low glycaemic index diet was accompanied by a small decrease in liver fat. In this study, the dietary GI was estimated on the basis of the GI tables provided by Brand-Miller’s group [26]. Further work is needed to identify the potential impact of such differences on insulin resistance.

The SACN report [1] concluded that lower glycaemic index diets were associated with reduced risk of cardiovascular disease, but it is noticeable that most of the studies included in this meta-analysis also appeared in the analysis of associations with fibre intake, and clearly further work is needed to establish whether the glycaemic characteristics or some features of the fibre content are responsible for the potential health benefits. Care must also be taken in promoting low glycaemic index per se, because the glycaemic index of fructose is very low and increasing the fructose content would reduce a food’s glycaemic index.

## Conclusions

There is an association between diets high in sugars (predominantly sucrose) and risk of disease, and experimental studies have shown that high intakes of fructose (over 100 g/d) can reduce insulin sensitivity, although somewhat lower intakes may affect serum TG. The mechanisms for such associations or effects have not been convincingly demonstrated. However, it remains to be seen whether it will be possible to unravel these mechanisms in the current climate in which marked decreases in sucrose/fructose intakes are being promoted as key public health strategies.

Overconsumption of fructose, as a contributor to an excessive energy intake is linked with increased liver and muscle fat contents, but a similar effect is seen with glucose and thus it may be more linked to carbohydrate per se, or possibly just to energy, overconsumption. Future work with overfeeding of fat is needed to explore this further.
